# Megalin: a Novel Determinant of Renin-Angiotensin System Activity in the Kidney?

**DOI:** 10.1007/s11906-020-01037-1

**Published:** 2020-03-14

**Authors:** Yuan Sun, Xifeng Lu, A. H. Jan Danser

**Affiliations:** 1 Department of Internal Medicine, Division of Pharmacology and Vascular Medicine, Erasmus MC, room EE1418b, Wytemaweg 80, 3015 CN Rotterdam, The Netherlands; 2 Department of Physiology, Shenzhen University Health Science Center, Shenzhen University, Shenzhen, China; 3 Translational Medicine Collaborative Innovation Center, The Second Clinical Medical College (Shenzhen People’s Hospital) of Jinan University, Shenzhen, China

**Keywords:** Megalin, V-ATPase, Chloride channel, Renin, Prorenin, Angiotensinogen, Proteinuria

## Abstract

**Purpose of Review:**

Megalin is well known for its role in the reabsorption of proteins from the ultrafiltrate. Recent studies suggest that megalin also reabsorbs renin and angiotensinogen. Indeed, without megalin urinary renin and angiotensinogen levels massively increase, and even prorenin becomes detectable in urine.

**Recent Findings:**

Intriguingly, megalin might also contribute to renal angiotensin production, as evidenced from studies in megalin knockout mice. This review discusses these topics critically, concluding that urinary renin-angiotensin system components reflect diminished reabsorption rather than release from renal tissue sites and that alterations in renal renin levels or megalin-dependent signaling need to be ruled out before concluding that angiotensin production at renal tissue sites is truly megalin dependent.

**Summary:**

Future studies should evaluate megalin-mediated renin/angiotensinogen transcytosis (allowing interstitial angiotensin generation), and determine whether megalin prefers prorenin over renin, thus explaining why urine normally contains no prorenin.

## Introduction

It is generally believed that renin-angiotensin system (RAS) activity in the kidney is independent of RAS activity in the circulation. Indeed, angiotensin (Ang) II levels in the kidney are several orders of magnitude higher than the levels of Ang II in blood and cannot be explained by uptake from the circulation [1, 2]. Virtually all renal Ang II is cell associated, either bound to Ang II type 1 (AT_1_) receptors on the cell surface or present intracellularly [3]. Studies in AT receptor knockout animals revealed that the intracellular presence of Ang II is dependent on AT_1_ receptor-mediated internalization, i.e., there is no evidence for intracellular Ang II generation [4]. Renal angiotensin generation therefore most likely occurs in the interstitial space. Remarkably, despite evidence for local angiotensinogen synthesis in the kidney, such generation, like circulating angiotensin generation, depends on liver-derived angiotensinogen [5, 6, 7•, 8]. With an identical substrate source, opposing changes in RAS activity in blood and kidney must be due to other RAS regulatory mechanisms, like the (local) release of renin and its precursor prorenin, the uptake of angiotensinogen from blood (possibly involving more than simple diffusion), and/or local alterations in ACE activity. For instance, in diabetes, the circulating RAS is suppressed, while renal RAS activity is increased [9]. This has been attributed to prorenin synthesis by the principal cells of the collecting duct, evidenced by increased renin activity in the collecting duct and elevated urinary renin levels in this condition [10–12]. However, a recent study in diabetic mice challenged this view and concluded that the increase in collecting duct renin under diabetic conditions reflects renin binding and not local synthesis [13••]. Moreover, the elevated urinary renin levels were due to enhanced glomerular filtration of plasma renin in combination with incomplete reabsorption by megalin, i.e., they did not reflect renin release from renal tissue sites (like the collecting duct). Surprisingly, megalin knockout even affected renal angiotensin generation [14••, 15•]. This review describes the role of megalin as a novel determinant of renal and urinary RAS activity.

## What Is Megalin?

Megalin is a single transmembrane protein consisting of 4655 amino acids with a molecular weight of 600 kDa. It has a large extracellular domain, a transmembrane region, and a small intracellular tail of 209 amino acids. It was originally known as glycoprotein 330 [16]. Megalin belongs to the low-density lipoprotein receptor (LDLR) superfamily [17] and contains four regions that consist of cysteine-rich complement-type repeats, four regions that consist of growth factor repeats spaced by eight YWTD repeats, and a single epidermal growth factor (EGF)-like repeat in its extracellular juxtamembrane region. The regions containing complement-type repeats are believed to be important for ligand binding, while the regions containing growth factor repeats are likely required for pH-dependent ligand dissociation in the endosomal compartment [18, 19]. The overall structure of megalin resembles LDLR-related protein 1 (LRP1), and therefore, megalin is also known as LRP2 [17]. The very large extracellular domain allows megalin to bind multiple ligands [20], and until now, > 50 ligands for megalin have been described, including albumin, insulin, insulin-like growth factor, lipoproteins, and drugs like aprotinin and polymyxin B [18, 21, 22].

Although megalin was initially identified as an antigen in glomerular podocytes, the expression of megalin in the glomerulus is negligible versus that in the proximal tubule [16, 18, 23–26]. Outside the kidney, megalin is also observed in absorptive epithelia in the lung, eye, gall bladder, placenta, and the (para)thyroid gland [18].

**Fig. 1:**
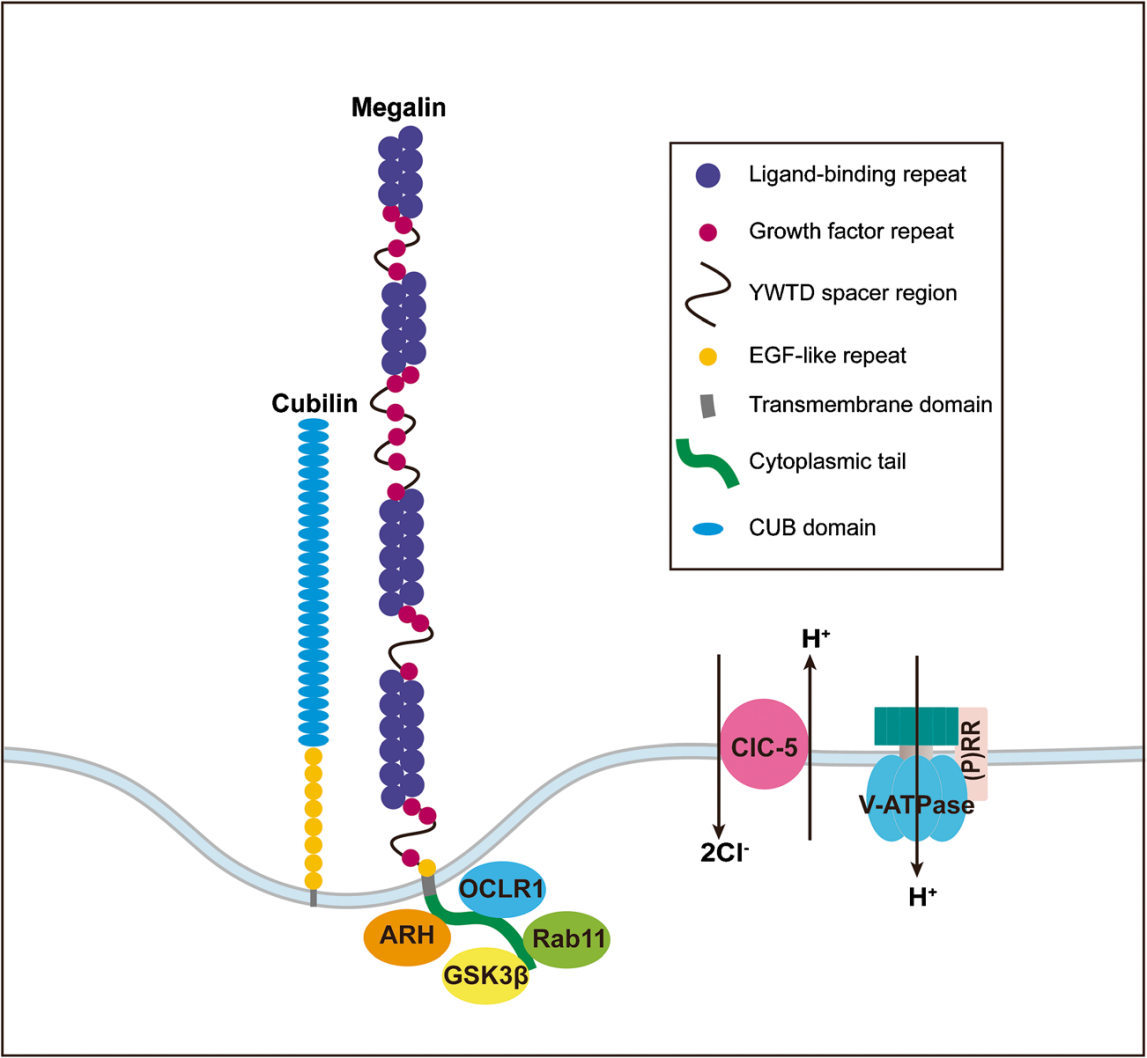
Megalin and its associated molecules in the proximal tubule. EGF, epidermal growth factor; OCRL1, oculocerebrorenal syndrome protein 1; ClC-5, voltage-gated chloride channel 5; ARH, autosomal recessive hypercholesterolemia; (P)RR, (pro)renin receptor; V-ATPase, vacuolar H^+^-ATPase; CUB, complement subcomponents C1r and C1s, fibropellin, and bone morphogenetic protein 1

Megalin forms a 1:1 complex with the extracellular protein cubilin on the apical plasma membrane of proximal tubule cells, and together, they mediate the endocytosis of ultrafiltrate proteins for subsequent lysosomal degradation and retrieval of their ligands and constituent amino acids into the blood. This prevents massive protein loss via urine. Megalin is a fast-recycling receptor with a long half-life, thereby making it ideal for reabsorption. Its endocytosis depends on the enzymatic activity of an inositol 5-phosphatase encoded by the gene oculocerebrorenal syndrome protein 1 (OCRL1) [27] (Fig. 1). The cytosolic tail of megalin is important for recycling and degradation [28]. Among others, it binds the small GTPase Rab11, and dominant negative mutations in Rab11 result in diminished apical delivery of megalin due to disturbed recycling [29]. Autosomal recessive hypercholesterolemia (ARH), an adaptor protein that binds megalin in a similar manner as the LDLR and clathrin, retains megalin in the recycling endosomes, so that it cannot reappear on the cell surface [30]. Furthermore, phosphorylation of the cytosolic tail by glycogen synthase kinase 3β also inhibits its recycling from endosomes back to the plasma membrane [31]. Fast recycling requires rapid disruption of the megalin-ligand complex through acidification in endosomes, a process depending on voltage-gated chloride channel 5 (ClC-5) and vacuolar H^+^-ATPase. Their colocalization is believed to allow ClC-5 to move in chloride as a counter-ion to the proton that is pumped by the vacuolar H^+^-ATPase [32, 33], thus ensuring optimal acidification.

### Genetic Mutations Affecting Megalin

LRP2 mutations, as occurring in the Donnai-Barrow/Facio-Oculo-Acoustico-Renal syndrome, result in impaired megalin function. Patients display proteinuria [34], hypercalciuria, nephrocalcinosis, nephrolithiasis, and focal segmental glomerulosclerosis [35]. Genome-wide association studies confirmed the link between LRP2 and proteinuria [36]. Interestingly, patients with mutations in the genes encoding for OCRL1 (Lowe syndrome) and ClC-5 (Dent’s disease) also display megalin dysfunction and proteinuria [37–39]. This can be explained based on the importance of OCRL1 and ClC-5 for megalin endocytosis and endosomal acidification, respectively (Fig. 1).

### Megalin and Kidney Disease

Megalin knockout mice display increased albumin excretion [40] and are protected against kidney damage resulting from a high-fat diet [24]. Similar protective effects are derived from megalin inhibition (e.g., with cilastatin) in relationship to acute kidney injury due to the uptake of nephrotoxic drugs like vancomycin and cisplatin [41]. Anti-brush border antibody disease or anti-LRP2 nephropathy involves the deposit of large amounts of circulating IgG through megalin in the renal tubule. This might explain why autoantibodies induce acute kidney injury [42]. Excess albumin, as occurring in patients with chronic kidney disease (CKD), directly contributes to the development and progression of CKD by inducing tubulointerstitial inflammation and fibrosis in a megalin-dependent manner [43]. Urinary excretion of megalin associates with renal oxidative stress in CKD [44, 45], while megalin excretion in both urine and urinary extracellular vesicles correlates with the progression of albuminuria in type 2 diabetes mellitus [46, 47]. The latter implies that urinary megalin could be a biomarker for diabetic nephropathy. Yet, renal megalin expression is decreased in diabetes [13••], although increases have been observed in the early stage of type 2 diabetes [48, 49]. Among the factors that lower megalin is Ang II [50].

### Megalin and Urinary RAS Components

Both renin and angiotensinogen can be detected in urine, albeit at very low levels (a few percent or less) when expressed as a percentage of their concomitant plasma concentrations [12, 51]. Prorenin is undetectable in urine [12]. The latter is not due to prorenin-renin conversion in urine [12]. Remarkably, renin and angiotensinogen massively rise (up to 40-fold) in urine of patients with Dent’s disease or Lowe syndrome [52], and the same occurs in urine of megalin knockdown mice [15•], or after megalin inhibition with lysine [53]. In fact, prorenin can also be detected in the urine of patients with Dent’s disease or Lowe syndrome. From these data, it can be concluded that RAS components are present in the ultrafiltrate, but normally are largely (> 95%) reabsorbed in a megalin-dependent manner [15•, 52–54], and possibly near-completely in the case of prorenin. Indeed, all 3 RAS components are filtered through the glomerulus and co-localize with megalin in the kidney [49, 54]. In fact, since both renin and prorenin are smaller than albumin, relatively larger amounts of renin and prorenin will pass the glomerulus in comparison with albumin. Not surprisingly, the glomerular sieving coefficients of renin, prorenin, and angiotensinogen increase after damaging the glomerular filtration barrier [52], e.g., in diabetes. Moreover, in mice, the rise in urinary renin after lysine was smaller under diabetic conditions than under normal conditions (≈ 10-fold versus ≈ 100-fold) [13••]. This is suggestive for disturbed tubular reabsorption. Therefore, the elevated urinary RAS component levels in diabetes do not reflect release from renal (tubular) tissue sites but are the consequence of enhanced filtration from blood plasma in combination with reduced reabsorption [13••]. One more condition where urinary renin, prorenin, and angiotensinogen levels are unexpectedly high is preeclampsia [55]. Like in diabetes, this may reflect enhanced filtration; to what degree megalin is downregulated in this condition is still unknown. It is also unknown whether megalin-dependent reabsorption is different for renin and prorenin.

### Megalin and Renal Angiotensin Production

After establishing that renal angiotensin generation depends on liver-derived angiotensinogen, while renal angiotensinogen staining was limited to proximal tubule cells expressing megalin, Matsusaka and colleagues studied whether megalin is a determinant of angiotensin production at renal tissue sites [14••]. It was observed that proximal tubule-specific knockout of megalin in mice did not affect renal Ang II levels, although such knockout did massively increase urinary angiotensinogen levels. Yet, when inducing podocyte injury with the immunotoxin LMB2, the well-known rise in renal angiotensinogen and Ang II levels no longer occurred without tubular megalin. As a consequence, the Ang II-mediated Na^+^ reabsorption via sodium-hydrogen exchanger 3 and the epithelial sodium channel were diminished, and thus the megalin knockout mice excreted 5 times more sodium after podocyte injury. In contrast with these findings, Ye et al. observed a profound (> 70%) drop in renal Ang II levels in wild type mice after megalin suppression based on the application of megalin antisense oligonucleotides (ASO) [15•]. The ASO approach did not affect circulating Ang II and resulted in the expected huge increase in urinary renin and angiotensinogen. Yet, even more surprising, megalin ASO reduced the atherosclerotic lesions in LDLR KO mice fed a high-fat diet. This suggests that renal Ang II, generated in a megalin-dependent manner, contributes to atherosclerosis. However, an alternative explanation might be that the ASO approach additionally interfered with megalin at extrarenal sites (as opposed to the proximal tubule-specific knockout applied by Matsusaka et al. [14••]), and that this underlies the beneficial effect on atherosclerosis. Indeed, megalin has been reported to contribute to Ang II internalization and signaling [50, 56] and binds lipoproteins. Ideally therefore, these data are confirmed in the proximal tubule-specific knockout model. With regard to renal angiotensin generation, both studies imply that this is reduced without megalin, either selectively under pathological conditions (following podocyte injury) or possibly already under healthy conditions. Here it is important to note that we do not know the renal renin levels in these studies, and thus a final possibility is that megalin knockout has altered these levels (although it did not affect renin gene expression). In fact, under most circumstances, it is variation in renin and not variation in angiotensinogen, which underlies variations in renal angiotensin generation, and even an angiotensinogen suppression of > 95% can be matched by a rise in renin [7•]. Indeed, renin rises of > 100-fold are easily achievable [57], and renal Ang II levels are unaltered when angiotensinogen is suppressed by 98% [7•].

**Fig. 2:**
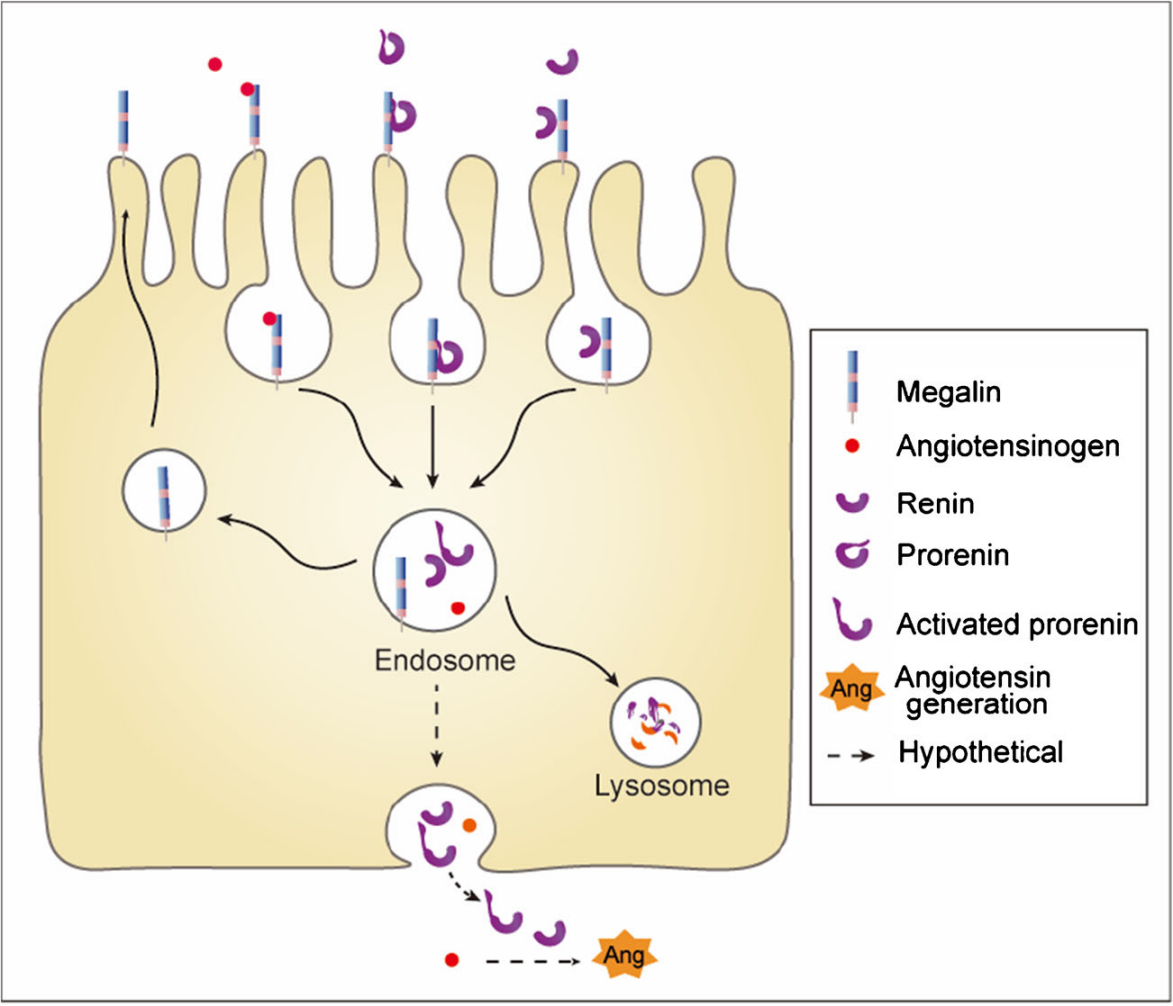
Megalin-mediated endocytosis of prorenin, renin, and angiotensinogen, resulting in lysosomal degradation and/or transcytosis and subsequent release into the renal interstitium, allowing angiotensin generation. The low pH in endosomes may result in non-proteolytic prorenin activation (i.e., removal of the prosegment from the enzymatic cleft, resulting in “open,” active prorenin)

When proposing a role for megalin in renal angiotensin generation, the question is how exactly this might occur. Obviously, endocytosis should then not result in angiotensinogen destruction. According to Wilson et al. [58], internalized angiotensinogen traffics intact to the mitochondria in isolated proximal tubules. However, given the absence of intracellular angiotensin generation [4], the implications of this finding are unclear. An attractive hypothesis would be that angiotensinogen is transcytosed to the basolateral membrane and released into the renal interstitium (Fig. 2). Such megalin-dependent transcytosis has been observed for albumin in rats [59, 60] and even for angiotensinogen in opossum kidney cells [54]. Diffusion of hepatic angiotensinogen from the circulation into the interstitial space has been reported decades ago as the most important, if not only, source of tissue angiotensin production [61]. It seems unlikely that the minute amounts of angiotensinogen in the ultrafiltrate under healthy conditions would contribute significantly to the renal interstitial angiotensinogen levels. However, under conditions of excessive protein/angiotensinogen leakage, assuming that megalin-mediated transcytosis takes place, it may indeed increase interstitial angiotensinogen and contribute to renal angiotensin production. In the case of prorenin, the acidic pH in the endosomes might even lead to prorenin activation, since a low pH results in non-proteolytic removal of the prosegment from the enzymatic cleft [62]. Yet, in previous studies, we were unable to show release of activated prorenin from prorenin-synthesizing cells, possibly because activated prorenin rapidly returns to the non-activated state in a neutral pH environment [63].

### Megalin and the (Pro)Renin Receptor

Numerous studies have investigated the so-called (pro)renin receptor as a novel component of the RAS over the last 2 decades. Although this receptor was initially reported to bind and activate prorenin [64], later studies revealed that the prorenin levels required to allow such interaction are many orders above its in vivo levels, even under pathological conditions [65, 66]. Hence, this concept is now being abandoned, also because it has become clear that the (pro)renin receptor is an accessory protein of vacuolar H^+^-ATPase (also known as ATP6AP2), and thus has important functions beyond the RAS, like facilitating vesicle trafficking, intracellular signaling, and lipid metabolism [67–70]. As such, it may also contribute to megalin sorting and recycling, since it determines endosomal acidification in concert with ClC-5 (Fig. 1). In agreement with this concept, ATP6AP2 inhibition impaired megalin protein regulation in Drosophila epithelial cells [71]. Thus, via megalin, the (pro)renin receptor may display a relationship with renin and prorenin.

## Conclusions and Remaining Questions

Megalin is a multi-ligand receptor mainly (but not exclusively!) expressed in the kidney, determining protein reabsorption in the proximal tubule. Both genetic mutations and kidney disease (e.g., diabetes, preeclampsia) affect its function and expression, thus contributing to kidney pathology. Since megalin also binds RAS components, an intriguing new concept is that it might even play a role in angiotensin generation at renal tissue sites. If so, its internalization should not result in destruction, but, for instance, via transcytosis, result in angiotensinogen release into the renal interstitial space. Such release would obviously depend on the level of angiotensinogen in the ultrafiltrate (reflecting podocyte injury) and tubular megalin expression, thus explaining why renal angiotensin generation may not always occur in concert with angiotensin generation in the circulation. Furthermore, megalin determines the level of RAS components in urine, and in the absence of megalin (like in Dent’s disease or Lowe syndrome), urinary renin and angiotensinogen levels can easily rise by 2 orders of magnitude [52]. As such, urinary renin and angiotensinogen components do not reflect release from renal tissue sites (like the collecting duct) but rather diminished uptake of filtered RAS components. Given the virtual absence of prorenin (despite its filtration) in urine, except under conditions where megalin function is disturbed, it seems that megalin prefers prorenin over renin. Future studies should now address this concept, e.g., making use of megalin-expressing cells like proximal tubule epithelial cells and Brown Norway Rat yolk sac epithelial cells. Such studies might also shed light on RAS component transcytosis. Additionally, in vivo renal angiotensin generation should be verified in the presence or absence of megalin and during inhibition of hepatic angiotensinogen synthesis, ruling out that alterations in renal renin levels and/or megalin-dependent signaling underlie the observed changes in kidney Ang II levels after megalin knockout [14••, 15•]. Knowledge on megalin expression in preeclampsia might shed light on the occurrence of prorenin in urine of women with preeclampsia [55]. Finally, we need to know what determines renin uptake in the collecting duct, reported recently by Tang et al. [13••], given that megalin is unlikely to occur in significant amounts at this location. Here, the link between megalin and the (pro)renin receptor may be of particular interest.
